# Downregulation of the long noncoding RNA GAS5-AS1 contributes to tumor metastasis in non-small cell lung cancer

**DOI:** 10.1038/srep31093

**Published:** 2016-08-04

**Authors:** Ying Wu, Hui Lyu, Hongbing Liu, Xuefei Shi, Yong Song, Bolin Liu

**Affiliations:** 1 Department of Respiratory Medicine, Jinling Hospital, Nanjing University School of Medicine, Nanjing, China; 2 Department of Pathology, School of Medicine, University of Colorado Anschutz Medical Campus, Aurora, CO, USA; 3 Department of Respiratory Medicine, Huzhou Central Hospital, Huzhou, China

## Abstract

Long noncoding RNA (lncRNA) plays pivotal roles in cancer development. To date, only a small number of lncRNAs have been characterized at functional level. Here, we discovered a novel lncRNA termed GAS5-AS1 as a tumor suppressor in non-small cell lung cancer (NSCLC). The expression of GAS5-AS1 in NSCLC tumors was much lower than that in the adjacent normal lung tissues. The reduced GAS5-AS1 was significantly correlated with larger tumors, higher TNM stages, and lymph node metastasis in NSCLC patients. While ectopic expression or specific knockdown of GAS5-AS1 had no effect on proliferation, cell cycle progression, and apoptosis, it dramatically decreased or increased, respectively, NSCLC cell migration and invasion. Overexpression of GAS5-AS1 in NSCLC cells reduced a cohort of molecules (ZEB1, N-cadherin, Vimentin, and/or Snail1) critical for epithelial-mesenchymal transition (EMT). Furthermore, the DNA demethylating agent 5-aza-2-deoxycytidine failed to upregulate GAS5-AS1 in NSCLC cells, whereas the pan-HDAC inhibitors panobinostat and SAHA significantly induced GAS5-AS1 in a dose-dependent manner. In addition, GAS5-AS1 can be upregulated by specific knockdown of HDAC1 or HDAC3. Collectively, our data suggest that histone modifications play a major role leading to epigenetic silencing of *GAS5-AS1* in NSCLC and subsequently promote tumor metastasis via upregulation of several key EMT markers.

Lung cancer is the leading cause of cancer-related death globally^1^. Despite tremendous efforts to reduce lung cancer deaths, the prognosis of lung cancer remains poor, with a 5-year survival rate of only 16%^2^. The current treatment for lung cancer patients who have been diagnosed at an early stage is surgical resection followed by chemotherapy. However, majority of the patients will eventually experience disease progression and require further treatment^3^. Although the platinum-based doublet regimens are the standard care of therapy for advanced non-small cell lung cancer (NSCLC), which includes adenocarcinoma and squamous cell carcinoma and accounts for approximately 85% of all lung cancer cases^4^,^5^, the patients often develop drug resistance and subsequent tumor metastasis. Metastasis is the major cause of morbidity and death in NSCLC, thus, understanding the molecular basis underlying NSCLC progression is crucial to improve the treatment and prognosis of patients with NSCLC.

Recent advent of techniques such as microarrays and high-throughput sequencing have led to the discovery that >90% of the total mammalian genome can be transcribed into many short or long noncoding RNAs^6^,^7^. Long noncoding RNAs (lncRNAs) are important class of the noncoding RNA family that are longer than 200 nt, with little protein-coding potential^8^. LncRNAs are often expressed in a spatial- and temporal-specific pattern^9^. Compared to the well-characterized microRNA (miRNA), the functions of lncRNAs have not been unraveled in detail. To date, a small number of lncRNAs have been shown to be involved in various tasks, such as chromatin modification, transcription or post-transcriptional regulation, organization of protein complexes, cell-cell signaling, and allosteric regulation of proteins^10^,^11^. Thus, lncRNAs can participate in diverse biological processes, including cell differentiation, modulation of apoptosis and invasion, reprogramming stem cell pluripotency, and parental imprinting^12^,^13^,^14^. Emerging evidence suggests that changes in lncRNA expression frequently occur in human cancers^15^,^16^. These findings underscore the significance to study the roles of tumor-associated lncRNAs, which may improve our understanding of the molecular basis of cancer initiation and progression. It has been shown that lncRNAs can function as oncogenes or tumor suppressors in a wide variety of human cancers^17^,^18^,^19^,^20^, including NSCLC^21^. However, the underlying mechanisms of lncRNA deregulation remain elusive. Methylation in the promoter region of lncRNAs has been found to result in aberrant expression of lncRNAs in human cancers^22^,^23^,^24^. Histone acetylation in the promoter region can also affect lncRNA transcriptional activation^25^,^26^. Thus, it indicated that epigenetic regulatory factors, including histone acetylation or DNA methylation, could manipulate the expression of lncRNAs.

Recent studies reveal that lncRNAs play a critical role in NSCLC pathogenesis^27^,^28^, providing a new avenue to explore the biology of this disease. We previously showed that the lncRNA GAS5 (growth arrest-specific transcript 5) was significantly downregulated in NSCLC tissues and cell lines; and elevated expression of GAS5 inhibited cell proliferation and induced apoptosis in NSCLC cells^29^. Nonetheless, as the antisense RNA of GAS5, GAS5-AS1’s expression and its biological role in NSCLC development and progression remains unknown. Here, we discovered that the reduced expression of GAS5-AS1 in NSCLC samples as compared to the adjacent normal lung tissues was significantly correlated with TNM stages, tumor size, and lymph node metastasis. We have also investigated the role of GAS5-AS1 in regulating NSCLC cell migration and invasion and explored the potential mechanism leading to downregulation of GAS5-AS1 in NSCLC.

## Results

### The expression of GAS5-AS1 is significantly downregulated in NSCLC cells

**Figure 1:**
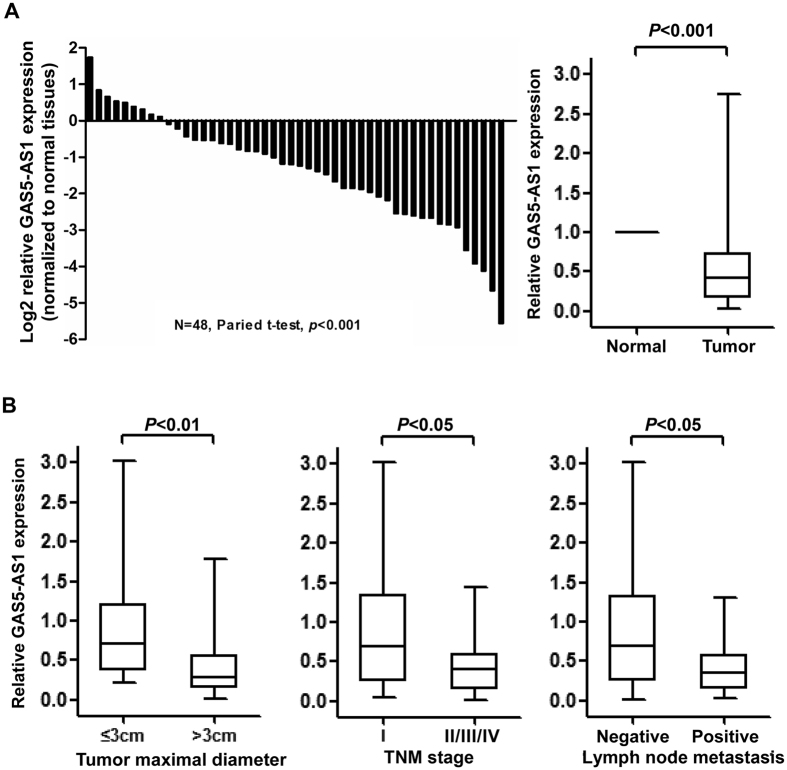
Significant downregulation of GAS5-AS1 in NSCLC tissues was correlated with tumor progression. (**A**) qRT–PCR analysis of the relative GAS5-AS1 expression in NSCLC tumors (n = 48) and their self-paired adjacent normal lung tissues (n = 48). (Left) The expression levels of GAS5-AS1 in NSCLC tumors are shown as log2-fold change to the adjacent normal lung tissues. (Right) The data are represented as a fold-change in the tumors relative to the normal tissues. The expression of GAS5-AS1 was significantly downregulated in NSCLC tumors as compared to the normal lung tissues. (**B**) The expression of GAS5-AS1 was significantly lower in larger tumors. The expression of GAS5-AS1 was significantly lower in the NSCLC patients with an advanced TNM stage than those with an early TNM stage, and was also significantly lower in the NSCLC patients with lymph node metastasis than those with no lymph node metastasis.

**Figure 2:**
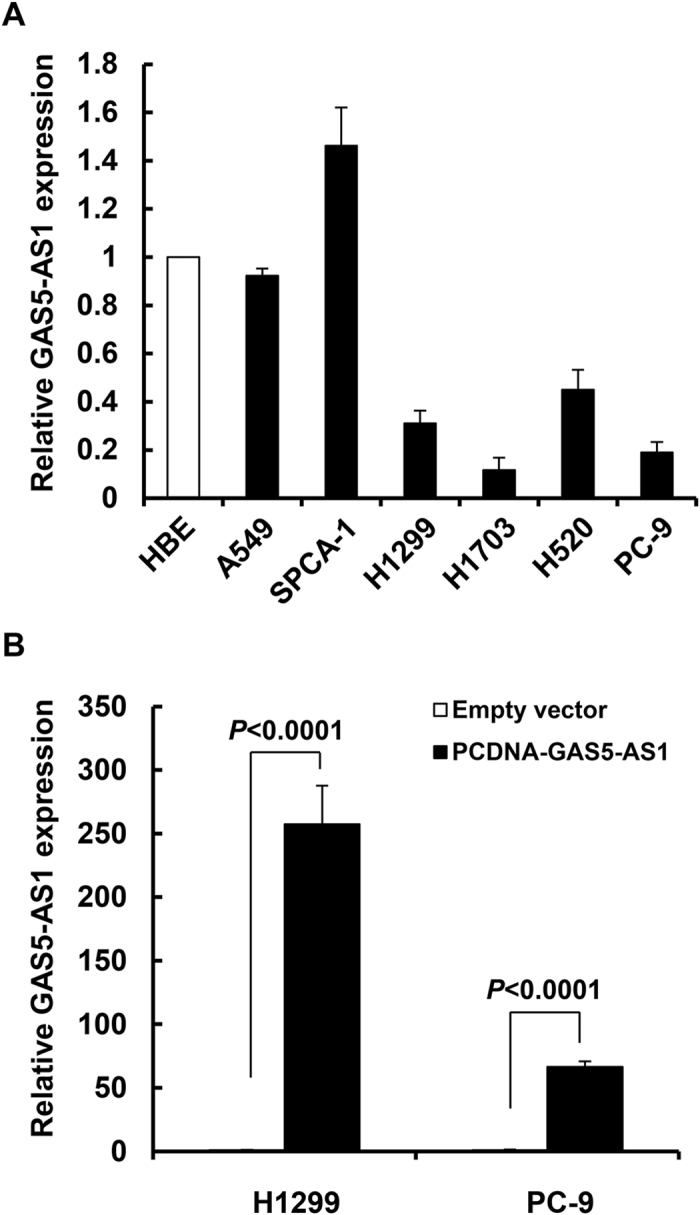
The expression of GAS5-AS1 was reduced in majority of the NSCLC cell lines tested. (**A**) qRT-PCR analysis of GAS5-AS1 expression in human bronchial epithelial cells (HBE) and six NSCLC cell lines (A549, SPC-A1, H1299, H1703, H520, and PC-9). (**B**) H1299 or PC-9 cells transfected with either pcDNA-GAS5-AS1 or empty vector were subjected to qRT-PCR measurement of GAS5-AS1 expression. Bars, SD. Data show a representative of three independent experiments.

To determine whether the lncRNA GAS5-AS1 plays a role in the development of NSCLC, we first performed qRT-PCR assays to measure the expression levels of GAS5-AS1 in 48 NSCLC tumors and their self-paired adjacent normal lung tissues. The expression of GAS5-AS1 was decreased in majority of the NSCLC tumors. Statistical analysis indicated that GAS5-AS1 was significantly downregulated in NSCLC tumors as compared to the normal tissues (Fig. 1A). Moreover, the reduced expression of GAS5-AS1 in NSCLC was associated with larger tumor size (>3 cm, *P* = 0.007), higher TNM stage (*P* = 0.012), and lymph node metastasis (*P* = 0.018) (Fig. 1B). However, the expression of GAS5-AS1 had no significant correlation with other parameters, such as age, gender, differentiation, smoking history, and histology type in NSCLC (Table 1). We next examined this lncRNA’s expression in a number of NSCLC cell lines, and discovered a much lower expression of GAS5-AS1 in 4 out of six NSCLC cell lines as compared to human bronchial epithelial (HBE) cells (Fig. 2A). Thus, significant downregulation of GAS5-AS1 is frequently observed in majority of the NSCLC tumors and cell lines.

### Ectopic expression or downregulation of GAS5-AS1 influences NSCLC cell migration and invasion without altering cell proliferation and apoptosis *in vitro*

**Figure 3:**
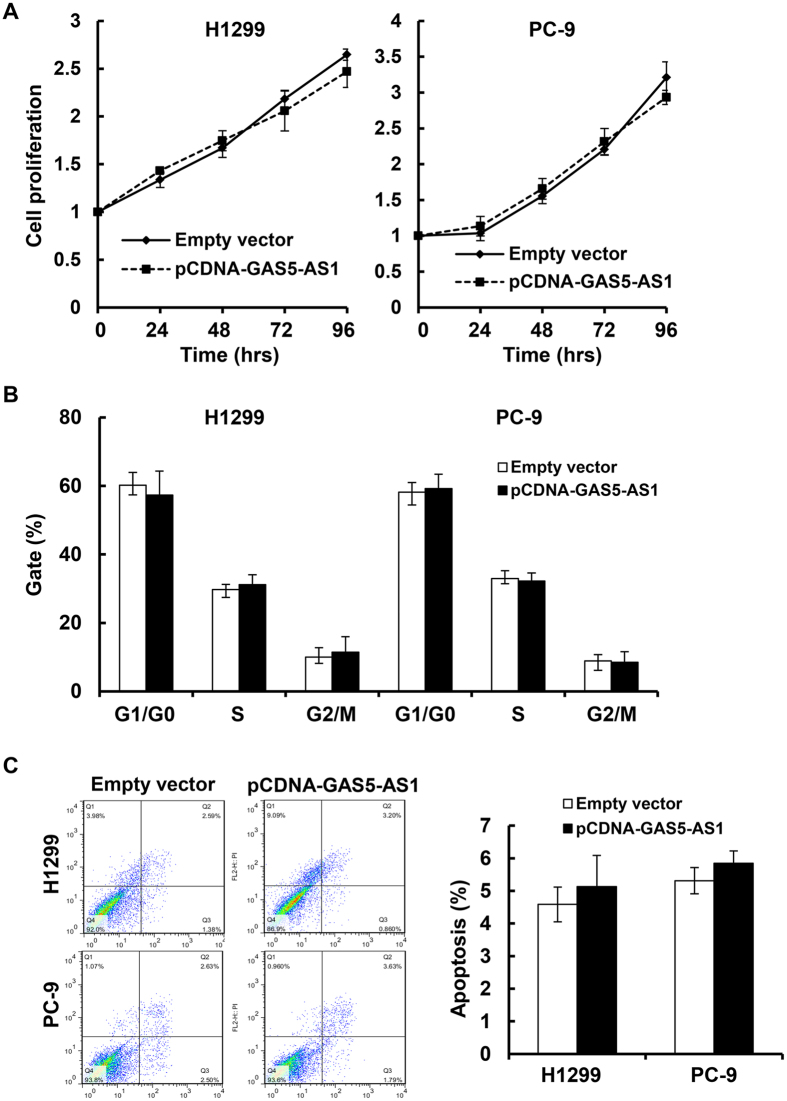
Ectopic expression of GAS5-AS1 did not alter NSCLC cell proliferation, cell cycle distribution, and apoptosis *in vitro*. H1299 or PC-9 cells were transfected with either pcDNA-GAS5-AS1 or empty vector. The cells were then collected and subjected to the following assays. (**A**) MTT assays were performed to determine the cell viability at indicated time points. (**B**) Cell cycle distribution was detected at 48 h following the transfections. The DNA contents were quantified by flow cytometric analysis. (**C**) At 48 h post-transfection, the cells were stained with FITC-Annexin V and propidium iodide (PI). The percentage of apoptotic cells was detected by flow cytometric analysis as described in the materials and methods. Bars, SD. Data show a representative of three independent experiments.

**Figure 4:**
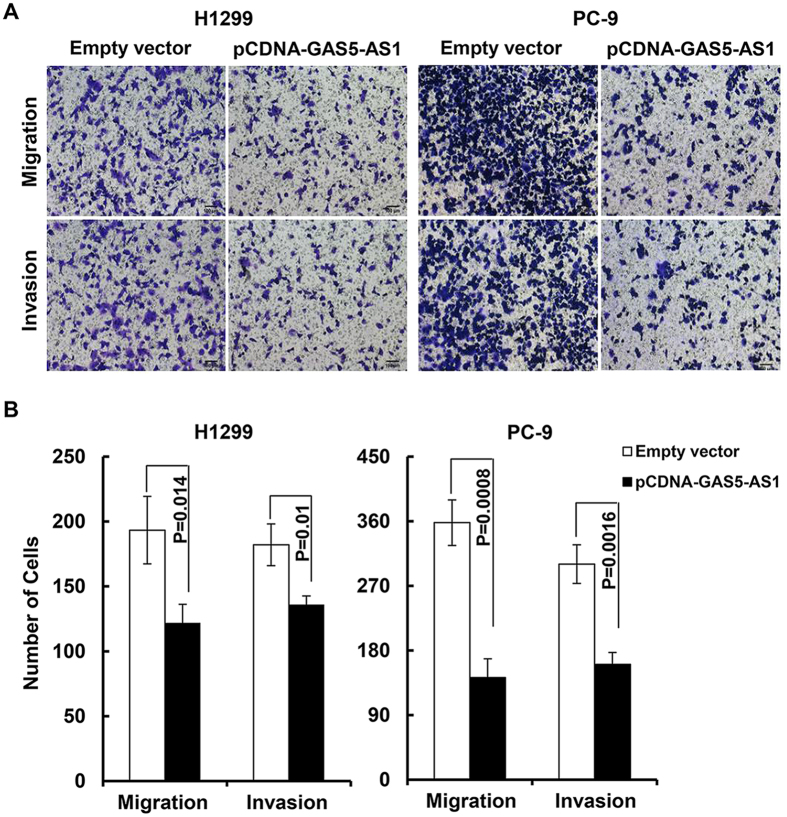
Overexpression of GAS5-AS1 significantly inhibited NSCLC cell migration and invasion. (**A**) H1299 or PC-9 cells were transfected with the empty vector or pcDNA-GAS5-AS1. Cell migration and invasion were determined with the Boyden chamber assays. Representative images of the transwell migration and invasion assays were shown. (**B**) Quantification analysis revealed that overexpression of GAS5-AS1 significantly inhibited migration and invasion in both H1299 and PC-9 cells. Bars, SD. Data show a representative of three independent experiments.

**Figure 5:**
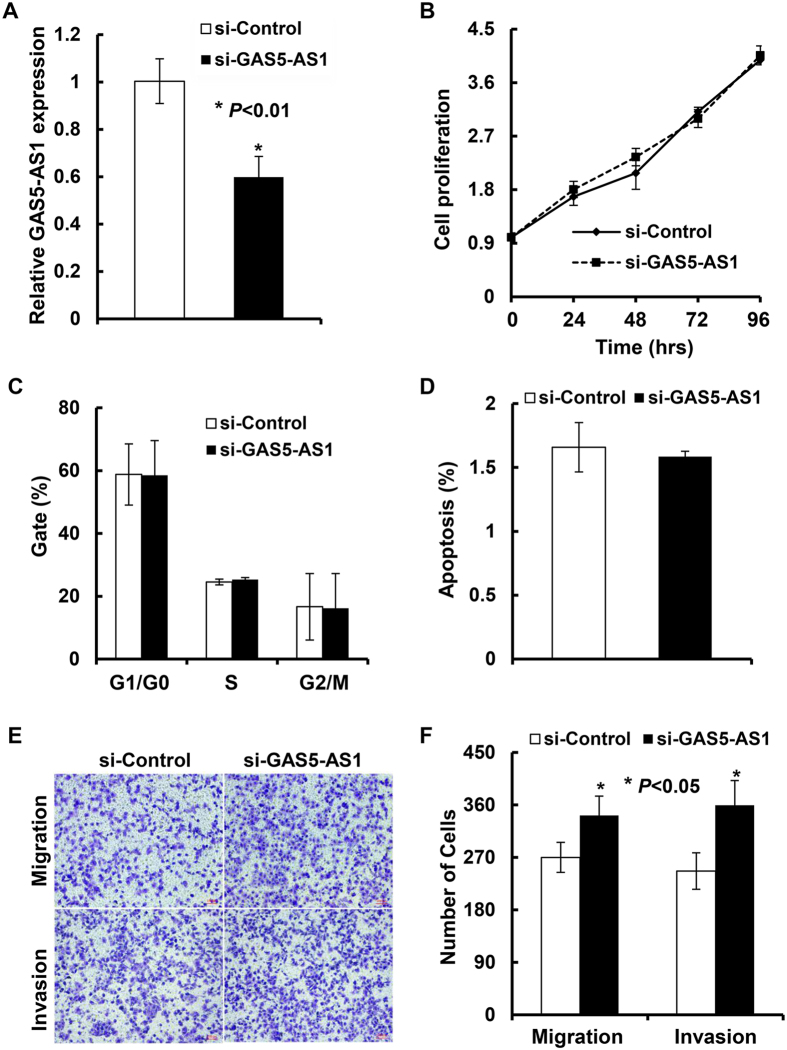
Specific knockdown of GAS5-AS1 expression in SPC-A1 cells increased cell migration and invasion without altering proliferation, cell-cycle progression, and apoptosis. SPC-A1 cells were transfected with either control siRNA (si-Control) or specific siRNA against GAS5-AS1 (si-GAS5-AS1), and then subjected to the following experiments: (**A**) GAS5-AS1 expression at 24 h post-transfection was measured by qRT-PCR analysis. (**B**) Cell proliferation was determined by an MTT assay at indicated time points. (**C**) Cell cycle distribution was analyzed by flow cytometry at 48 h following the transfection. (**D**) At 48 h post-transfection, the cells were stained with FITC-Annexin V and PI. The percentage of apoptotic cells was detected by flow cytometric analysis. (**E**,**F**) Transwell assays were performed to determine the migratory and invasive abilities of the cells. Bars, SD. Data show a representative of three independent experiments.

To assess the biological function of GAS5-AS1 in NSCLC, we first examined the impact of GAS5-AS1 expression on the proliferation and apoptosis of NSCLC cells. Overexpression of GAS5-AS1 was achieved via transient transfection of pCDNA-GAS5-AS1 into H1299 or PC-9 cells. The expression of GAS5-AS1 was significantly increased as compared to the same cells transfected with empty vector (Fig. 2B). However, ectopic expression of GAS5-AS1 did not impair the growth of H1299 and PC-9 cells as compared to the empty vector-transfected cells (Fig. 3A). Cell cycle analysis of the H1299 or PC-9 cells transfected with pCDNA-GAS5-AS1 or empty vector showed no significant alterations in the percentage cells of G1/G0, S, and G2/M phases (Fig. 3B). Additionally, we performed flow cytometric analysis of apoptotic cells with Annexin V staining, and found that the increased GAS5-AS1 expression in H1299 or PC-9 cells did not induce apoptosis (Fig. 3C). To further assess the importance of GAS5-AS1 in NSCLC progression, we wondered whether GAS5-AS1 expression might alter the metastatic potential of NSCLC cells. We thus performed Boyden chamber transwell assays to examine cell migration and invasion. The increased GAS5-AS1 expression significantly impeded the migration of H1299 and PC-9 cells. Similarly, the invasiveness of H1299 and PC-9 cells-transfected with pCDNA-GAS5-AS1 was also dramatically reduced (Fig. 4), indicating that ectopic expression of GAS5-AS1 inhibited migratory and invasive phenotype of NSCLC cells. Conversely, we performed reciprocal experiments to examine the effects of GAS5-AS1 knockdown on cell proliferation and apoptosis as well as migration and invasion in SPC-A1 cells, which express high levels of GAS5-AS1 (Fig. 2A). Significant downregulation of GAS5-AS1 in SPC-A1 cells upon specific siRNA transfection was confirmed by qRT-PCR analysis (Fig. 5A). Compared to the negative control siRNA-transfected cells, specific knockdown of GAS5-AS1 did not change cell proliferation (Fig. 5B), cell cycle progression (Fig. 5C), and apoptosis (Fig. 5D). In contrast, specific knockdown of GAS5-AS1 in SPC-A1 cells significantly increased cell migration and invasion (Fig. 5E,F). Taken together, our data demonstrate that altered expression of GAS5-AS1 does not affect NSCLC cell proliferation and apoptosis *in vitro*. The biological function of GAS5-AS1 in NSCLC may influence tumor metastasis via regulating cell migration and invasion.

### Elevated expression of GAS5-AS1 seems to inhibit the expression of several EMT markers in NSCLC cells

**Figure 6:**
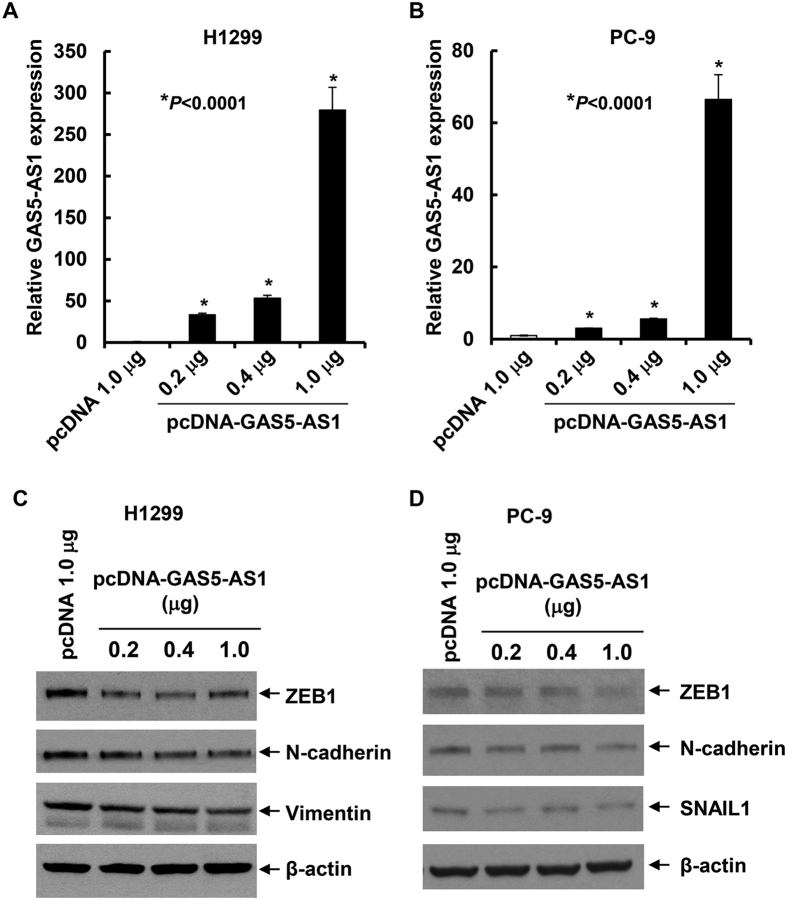
Increased GAS5-AS1 reduced expression of several EMT markers in NSCLC cells in a dose-dependent manner. H1299 and PC-9 were transfected with the empty vector (pcDNA) or pcDNA-GAS5-AS1 at the indicated concentrations for 48 h. (**A**,**B**) Half of the cells were collected and subjected to total RNA extraction. qRT-PCR analyses were performed to detect the expression levels of GAS5-AS1. (**C**,**D**) The other half of the cells were subjected to western blot analyses with specific antibody. The expression levels of ZEB1, N-cadherin, and Vimentin were determined in H1299 cells, whereas ZEB1, N-cadherin, and SNAIL1 were analyzed in PC-9 cells.

Since epithelial-mesenchymal transition (EMT) is a key step in the process of tumor infiltration or progression, and it is a critical mechanism underlying metastasis and cancer cell invasion^30^,^31^. We next sought to explore whether there was any interaction between EMT-related markers and GAS5-AS1 expression. The expression levels of GAS5-AS1 in H1299 or PC-9 cells gradually increased via transfection with different concentrations of pcDNA-GAS5-AS1 (Fig. 6A,B). Overexpression of GAS5-AS1 in H1299 cells reduced ZEB1, N-cadherin, and Vimentin in a dose-dependent manner (Fig. 6C), whereas the expression of ZEB1, N-cadherin, and Snail1 protein was gradually decreased upon ectopic expression of GAS5-AS1 in PC-9 cells (Fig. 6D). Collectively, our data suggest that GAS5-AS1 modulates the metastatic potential of NSCLC cells in part through its influence on expression of several key EMT markers.

### HDAC inhibitors induce expression of GAS5-AS1 in NSCLC cells

**Figure 7:**
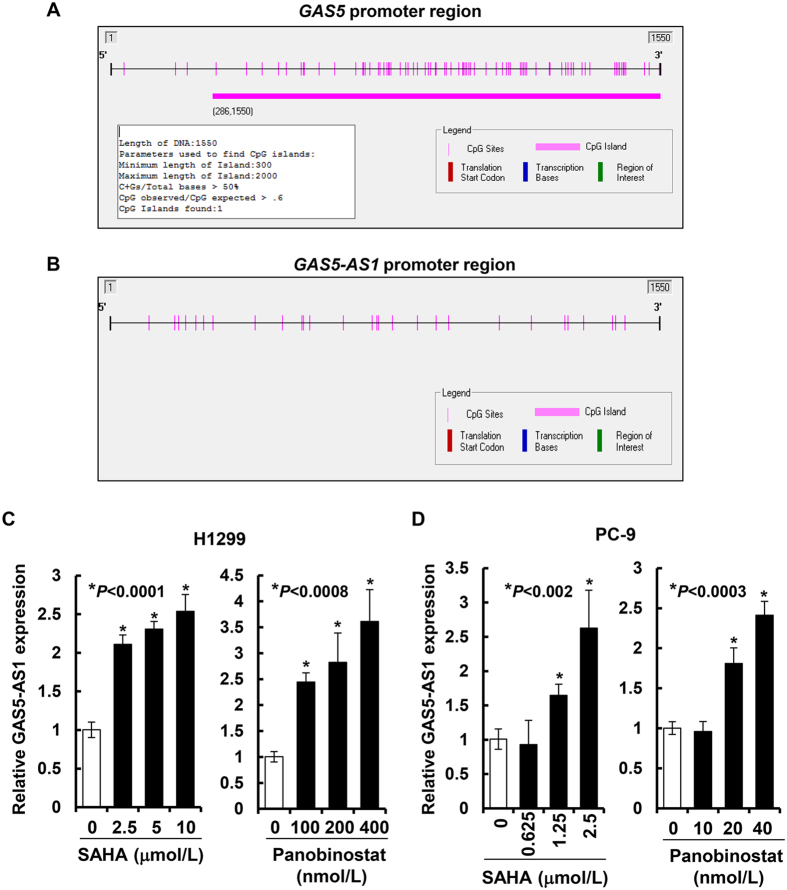
Histone acetylation seemed to regulate the expression of GAS5-AS1 in NSCLC cells. (**A**,**B**) Methyl Primer Express software (v1.0) was used to detect CpG islands in the promoter regions of GAS5 and GAS5-AS1. (**C**,**D**) H1299 or PC-9 untreated or treated with the indicated concentrations of SAHA or panobinostat for 24 h were collected and subjected to total RNA extraction. The expression levels of GAS5-AS1 were determined by qRT-PCR. Bars, SD. Data show a representative of three independent experiments.

We previously showed that promoter methylation seemed to contribute to the reduction of GAS5-AS1’s sister lncRNA GAS5 in NSCLC^29^. Indeed, bioinformatics analysis identified CpG islands in the *GAS5* promoter region (Fig. 7A). However, no CpG island was found in the promoter region of *GAS5-AS1* (Fig. 7B), suggesting that DNA methylation might not be the major mechanism resulting in downregulation of GAS5-AS1 in NSCLC. To verify our prediction, we treated H1299 and PC-9 cells with the demethylating agent decitabine at different concentrations. As expected, while decitabine was able to induce expression of GAS5, it had no significant effect on GAS5-AS1 expression (Supplementary Figure S1). Histone modification is another important mechanism controlling transcriptional activation of lncRNAs. Hence, we next investigated whether the aberrant expression of GAS5-AS1 in NSCLC was attributed to the changes of histone acetylation. H1299 and PC-9 cells were treated with the pan inhibitors of histone deacetylase (HDAC), SAHA or panobinostat. We discovered that the expression levels of GAS5-AS1 were significantly upregulated by both SAHA and panobinostat in a dose-dependent manner (Fig. 7C,D). To provide a direct evidence supporting the impact of histone modification on GAS5-AS1 expression in NSCLC cells, we performed additional experiments and found that specific knockdown of HDAC1 or HDAC3 with siRNA was able to significantly enhance the expression levels of GAS5-AS1 in H1299 (Supplementary Figure S2A) and PC-9 cells (Supplementary Figure S2B). Taken together, our data indicate that histone deacetylation, but not promoter methylation at least partially contributes to the downregulation of GAS5-AS1 in NSCLC.

## Discussion

LncRNAs have been shown to play a critical role in tumorigenesis, and contributes to a diverse of biological functions in human cancers^32^,^33^. To date, the roles of vast majority of lncRNAs in NSCLC initiation and progression are far from being fully elucidated. Tumor metastasis is the major cause of death in patients with NSCLC, thus understanding the molecular mechanism by which a specific lncRNA is involved in metastasis may provide novel opportunities to identify effective therapy against NSCLC. Recent studies suggest that several lncRNAs are dysregulated in multiple cancers, including NSCLC^17^,^34^,^35^. One of these is metastasis-associated lung adenocarcinoma transcript 1 (MALAT1), also known as nuclear-enriched abundant transcript 2 (NEAT2), which is a highly conserved nuclear lncRNA and a predictive marker for metastasis in lung cancer^17^,^28^. In the current study, we discovered that the lncRNA GAS5-AS1 was downregulated in NSCLC tumors as compared to the adjacent normal lung tissues, and the expression of GAS5-AS1 was significantly lower in NSCLC patients with later stages of tumors and lymph node metastasis. These findings indicate that GAS5-AS1 may function as a tumor suppressor in the modulation of NSCLC progression.

As the antisense RNA of GAS5, GAS5-AS1 (NCBI no. NR_037605.1) is a novel lncRNA transcript which maps on chromosome 1. Our recent studies revealed that GAS5 was also significantly downregulated in NSCLC tumors and cell lines^29^. While GAS5 was found to mainly inhibit proliferation and induce apoptosis in NSCLC cells, the role of GAS5-AS1 in NSCLC remained unknown. We thus investigated the effects of GAS5-AS1 overexpression on various biology aspects of NSCLC cells. Two NSCLC cell lines (H1299 and PC-9) that are commonly used and express low levels of GAS5-AS1 (Fig. 2A) were chosen to evaluate the effect of GAS5-AS1 on cell behavior. We observed that elevated expression of GAS5-AS1 did not alter cell proliferation and cell cycle progression, and had no influence on the cells undergoing apoptosis in the NSCLC cells tested (Fig. 3). Instead, ectopic expression of GAS5-AS1 led to a dramatic inhibition of migration and invasion in both H1299 and PC-9 cells (Fig. 4). Conversely, similar conclusions were also supported by the reciprocal experiments. Specific knockdown of GAS5-AS1 expression in SPC-A1 cells, which express high levels of GAS5-AS1 (Fig. 2A), significantly increased cell migration and invasion without affecting proliferation, cell cycle progression, and apoptosis (Fig. 5). Collectively, our data suggest that while both GAS5 and GAS5-AS1 play tumor suppressive roles in NSCLC development, these two lncRNAs function in a distinct way. i.e. GAS5 mainly inhibits NSCLC cell proliferation and induces apoptosis, whereas GAS5-AS1 is crucial to repress NSCLC cell migration and invasion. These novel findings inspire us to hypothesize that the NSCLC cells with low expression of both GAS5 and GAS5-AS1 may be more aggressive than those with low expression of either one of them. To test this hypothesis, further analyses of additional clinical samples of NSCLC patients are needed. Meanwhile, we are not sure whether co-transfection of GAS5 and GAS5-AS1 into the same NSCLC cell line may induce severe growth inhibition and/or apoptosis. We are currently performing the experiments in our laboratory.

To better understand the molecular basis responsible for the inhibition of migration and invasion mediated by GAS5-AS1 in NSCLC, we explored potential targets associated with cell migration and invasion. Elevated expression of GAS5-AS1 in H1299 and PC-9 cells clearly decreased ZEB1 and N-cadherin protein levels in a dose-dependent manner (Fig. 6C,D). The increased GAS5-AS1 also reduced vimentin in H1299 cells and decreased SNAIL1 in PC-9 cells. All of these are well-known markers of EMT^36^,^37^, an initial event to promote tumor cell invasion and metastasis^30^,^38^. A number of studies have revealed an associative relationship between EMT and lung cancer metastasis^39^,^40^. To the best of our knowledge, we are the first providing experimental data showing that GAS5-AS1 contributes to NSCLC cell migration and invasion at least partially through regulation of EMT. Nonetheless, detailed studies of the signaling pathway responsible for the biological functions of GAS5-AS1 in EMT are needed.

It has been reported that tumor suppressor genes are usually inactivated by genetic or epigenetic alterations in cancer cells^41^. The expression of ncRNAs can be modified by epigenetic factors, including DNA methylation and histone modification. A recent study of genome-wide DNA methylome analysis reveals that aberrant promoter methylation contributes to dysregulated ncRNAs in human breast cancer cells^42^. Some studies suggest that deregulation of histone acetylation and deacetylation also plays an important role in aberrant expression of lncRNAs, such as lncRNA-LET^26^. Moreover, lncRNA array analysis show that thousands of lncRNAs are regulated by the pan-HDAC inhibitor LBH589 (panobinostat) in wilms tumor cells^43^. The data we presented here highlight that GAS5-AS1 can be induced by two pan-HDAC inhibitors, panobinostat and SAHA (Fig. 7C,D). Moreover, specific knockdown of HDAC1 or HDAC3 also enhances expression of GAS5-AS1 in both H1299 and PC-9 cells (Supplementary Figure S2), suggesting that histone deacetylation may directly involve in regulation of GAS5-AS1 transcriptional activation. Interestingly, a CpG island was found in the promotor region of GAS5 (Fig. 7A), which could be upregulated by DNA demethylating agents^29^ and (Supplementary Figure S1). In contrast, there is no CpG island in the promotor region of GAS5-AS1 (Fig. 7B) and the DNA demethylating agent does not induce GAS5-AS1 expression in NSCLC cells (Supplementary Figure S1). Taken together, these data further our understanding of the epigenetic role in regulating lncRNA expression in NSCLC.

In summary, we discovered that GAS5-AS1 was downregulated in NSCLC tumors and the reduced expression of GAS5-AS1 significantly associated with larger tumors (>3 cm), higher TNM stage, and lymph node metastasis, Further studies showed that ectopic expression of GAS5-AS1 inhibited NSCLC cell migration and invasion partially through repression of several key EMT markers. It appeared that both GAS5-AS1 and its “sister” lncRNA GAS5 functioned as tumor suppressors in NSCLC, however, they acted via distinct mechanisms. GAS5 mainly inhibits NSCLC cell proliferation and induces apoptosis, whereas GAS5-AS1 is vital to repress NSCLC cell migration and invasion without altering proliferation/survival.

## Methods

### Patients and tissue samples

Forty eight paired NSCLC and the adjacent non-tumor lung tissues were obtained from patients who underwent surgery at Department of Thoracic Surgery, Jinling Hospital, Nanjing University School of Medicine, Nanjing, China, between November 2011 and September 2014. All patients were diagnosed with NSCLC according to histopathological features. The patients’ clinicopathological characteristics, including tumor-node-metastasis (TNM) staging, were summarized in Table 1. No preoperative adjuvant therapy was conducted. Following surgery, all the tissue samples were immediately frozen in liquid nitrogen and stored at −80 °C until required. The clinical samples were acquired with written informed consent from all of the participants following protocols approved by the Institutional Review Board of Nanjing University. All experimental procedures used in the study were carried out in accordance with the approved guidelines by the Institutional Review Board of Nanjing University.

### Cells and cell culture

Human NSCLC cell lines (A549, SPCA-1, H1299, H1703, H520, and PC-9) and the 16-HBE (HBE bronchial epithelial cells) were purchased from the Institute of Biochemistry and Cell Biology of the Chinese Academy of Sciences (Shanghai, China). A549, H1703, H520, and HBE cells were maintained in RPMI1640 medium supplemented with 10% fetal bovine serum (FBS). SPCA-1, H1299, and PC-9 cells were maintained in DMEM/F12 medium containing 10% FBS. All cell lines were free of mycoplasma contamination, which was determined by the MycoAlert™ Mycoplasma Detection Kit (Lonza Group Ltd. Basel, Switzerland) every three months. All cell lines were cultured in a 37 °C humidified atmosphere containing 95% air and 5% CO_2_ and were split twice a week.

### RNA extraction and quantitative real-time PCR (qRT-PCR)

Total RNA was extracted from frozen tissue or cultured cells using TRIZOL reagent (Invitrogen, Shanghai, China) following the manufacturer’s instructions. For detection of GAS5-AS1, the RNA was reverse-transcribed into cDNA using the PrimeScript RT reagent Kit (TaKaRa, Dalian, China). To quantify the expression levels of GAS5-AS1, qRT-PCR was performed using the FastStart Universal SYBR Green Master Mixes (Roche Diagnostics Corp., Indianapolis, IN) by a 7900 Fast Real-Time PCR system (Applied Biosystems, Foster City, CA). The expression of GAPDH was used for normalization. The specific primers were as follows: GAPDH sense 5′-GTCAACGGATTTGGTCTGTATT-3′, reverse 5′-AGTCTTCTGGGTGGCAGTGAT-3′; GAS5-AS1 sense 5′-TCCCAGCCTCAGACTCAACA-3′, reverse 5′-GTTTCATAGGCCCCTGTGCT-3′; GAS5 sense 5′-CTTGCCTGGACCAGCTTAAT-3′, reverse 5′-CAAGCCGACTCTCCATACCT-3′. HDAC1 sense 5′-GGAAATCTATCGCCCTCACA-3′, reverse 5′-AACAGGCCATCGAATACTGG-3′, HDAC3 sense 5′-TAGACAAGGACTGAGATTGCC-3′, reverse 5′-GTGTTAGGGAGCCAGAGCC-3′.

### Construction of the expression vector pcDNA-GAS5-AS1 and cell transfection

The GAS5-AS1 sequence was synthesized and subcloned into a pCDNA3.1 vector (Invitrogen, Shanghai, China). The pCDNA-GAS5-AS1 or empty vector was prepared using DNA Midiprep kits (Qiagen, Hilden, Germany) and transfected into H1299 or PC-9 cells. The empty pcDNA3.1 vector was used as the control. H1299 or PC-9 cells were cultured on six-well plates to confluency and transfected using FuGENE 6 transfection reagent (Roche Applied Science) according to the manufacturer’s instructions. The cells were harvested for qRT-PCR or western blot analysis at 48 h post plasmid transfection.

### Construction of siRNA and transfection of cell lines

The siRNA (small interfering RNA) sequences were as follows: GAS5-AS1 siRNA sense 5′-GCAGUGUUAAUGAAGCAAATT-3′, antisense 5′-UUUGCUUCAUUAACACUGCTT-3′; HDAC1 siRNA sense 5′-GCUCCUCUGACAAACGAAUTT-3′, antisense 5′-AUUCGUUUGUCAGAGGAGCTT-3′ HDAC3 siRNA sense 5′-GGUAGUGGACUUCUACCAATT-3′, antisense 5′-UUGGUAGAAGUCCACUACCTT-3′; negative control siRNA sense 5′-UUCUCCGAACGUGUCACGUTT-3′, antisense 5′-ACGUGACACGUUCGGAGAATT-3′. All the synthetic siRNAs were purchased from Genepharma (Genepharma, Shanghai, China). The siRNAs were transfected into cells, which were cultured on six-well plates, using Lipofectamine 2000 (Invitrogen, Shanghai, China) according to the manufacturer’s instructions.

### Cell proliferation assay

A cell proliferation assay was performed by use of MTT kit (Roche Applied Science) following the manufacturer’s protocol. The pCDNA-GAS5-AS1 or empty vector-transfected H1299 or PC-9 cells were allowed to grow in 96-well plates (3,000/well). Cell viability was documented every 24 h. All of the experiments were performed in quadruplicates.

### Analysis of apoptosis and cell cycle by flow cytometry

H1299 and PC-9 cells were harvested at 48 h post-transfection. Following double staining with FITC-Annexin V and propidium iodide (PI), cells were analyzed by flow cytometry (FACScan) using CellQuest software (BD Biosciences). Cells were categorized into viable cells, dead cells, early apoptotic cells, and late apoptotic cells. The ratio of apoptotic cells was compared to the control group from each experiment. For cell cycle analysis, the cells stained with PI were analyzed using the Cycle TESTTMPLUS DNA reagent kit (BD Biosciences) and FACScan. The proportion of cells in the G0/G1, S, and G2/M phases were counted and compared. All samples were tested in triplicates.

### Cell migration and invasion assays

For migration assays, after transfected for 48 h with pCDNA-GAS5-as1 or empty vector, 5 × 10^4^ cells in serum-free media were placed into the upper chamber of an insert (8-μm pore size; Millipore). For invasion assays, 1 × 10^5^ cells in serum-free medium were seeded into the upper chamber of an insert coated with Matrigel. Medium containing 10% FBS was placed over the lower chamber. After incubation for 24 h, we removed the upper layer of cells with cotton wool, and the cells on the lower surface were fixed in methanol and stained with 0.1% crystal violet, then imaged using an IX71 inverted microscope (Olympus, Tokyo, Japan). All experiments were performed in triplicates.

### Western blot analysis

Protein expression was determined by western blots as previously described^44^,^45^,^46^. In brief, equal amounts of total cell lysates were boiled in SDS-sample buffer, resolved by SDS–PAGE, transferred onto nitrocellulose membrane (Bio-Rad Laboratories, Hercules, CA), and then incubated with the primary antibody. After the blots were incubated with HRP-labeled 2^nd^ antibody (Jackson ImmunoResearch Laboratories, West Grove, PA), the signals were detected by the enhanced chemiluminescence reagents (GE Healthcare Bio-Sciences Corp., Piscataway, NJ). Antibodies against ZEB1, N-cadherin, Vimentin, and Snail1 (EMT Antibody Sampler Kit #9782) were purchased from Cell Signaling Technology (Beverly, MA). Antibody against β-actin was from Sigma-Aldrich (Sigma Co., St. Louis, MO).

### Statistical Analysis

Statistical analysis was performed using SPSS version 22 software (Chicago, IL). Student’s t-test (two-tailed) or one-way ANOVA were used to analyze the data. Statistical significance was set at a *P*-value less than 0.05.

## Additional Information

**How to cite this article**: Wu, Y. *et al*. Downregulation of the long noncoding RNA GAS5-AS1 contributes to tumor metastasis in non-small cell lung cancer. *Sci. Rep.* **6**, 31093; doi: 10.1038/srep31093 (2016).

## Supplementary Material

Supplementary Information

## Figures and Tables

**Table 1:** Correlation between GAS5-AS1 expression and clinicopathological parameters of NSCLC patients.

Characteristics	N of cases	Relative expression of GAS5-AS1		
		Low	High	*P*-value^a^
Age (years)				0.53
≤65	31	26	5	
>65	17	13	4	
Gender				0.218
male	36	30	6	
female	12	8	4	
Differentiation				0.485
well, moderate	27	21	6	
poor	21	18	3	
Tumor size (maximum diameter)				0.019*
≤3 cm	16	10	6	
>3 cm	32	29	3	
Smoking History				0.565
smokers	28	22	6	
never Smokers	20	15	6	
Histology type				0.39
adenocarcinoma	23	17	6	
squamous carcinoma	25	21	4	
Lymph node metastasis				0.022*
positive	27	25	2	
negative	21	14	7	
TMN stage				0.017*
I	18	11	7	
II/III/IV	30	27	3	

^a^Chi-square test.

^*^P < 0.05.
