# Supplementary material for: Downregulation of the long noncoding RNA GAS5-AS1 contributes to tumor metastasis in non-small cell lung cancer
Source: Sci Rep. 2016 Aug 4;6:31093. doi: 10.1038/srep31093 (PMC4973264; doi:10.1038/srep31093)
Supplement: Supplementary Information [file srep31093-s1.pdf]

Downregulation of the long noncoding RNA GAS5-AS1 contributes to tumor metastasis in non-small cell lung cancer

Ying Wu[1,2], Hui Lyu[2], Hongbing Liu[1], Xuefei Shi[3], Yong Song[1,*], and Bolin Liu[2,*]

[1]Department of Respiratory Medicine, Jinling Hospital, Nanjing University School of Medicine, Nanjing, China; [2]Department of Pathology, School of Medicine, University of Colorado Anschutz Medical Campus, Aurora, CO, USA; [3]Department of Respiratory Medicine, Huzhou Central Hospital, Huzhou, China

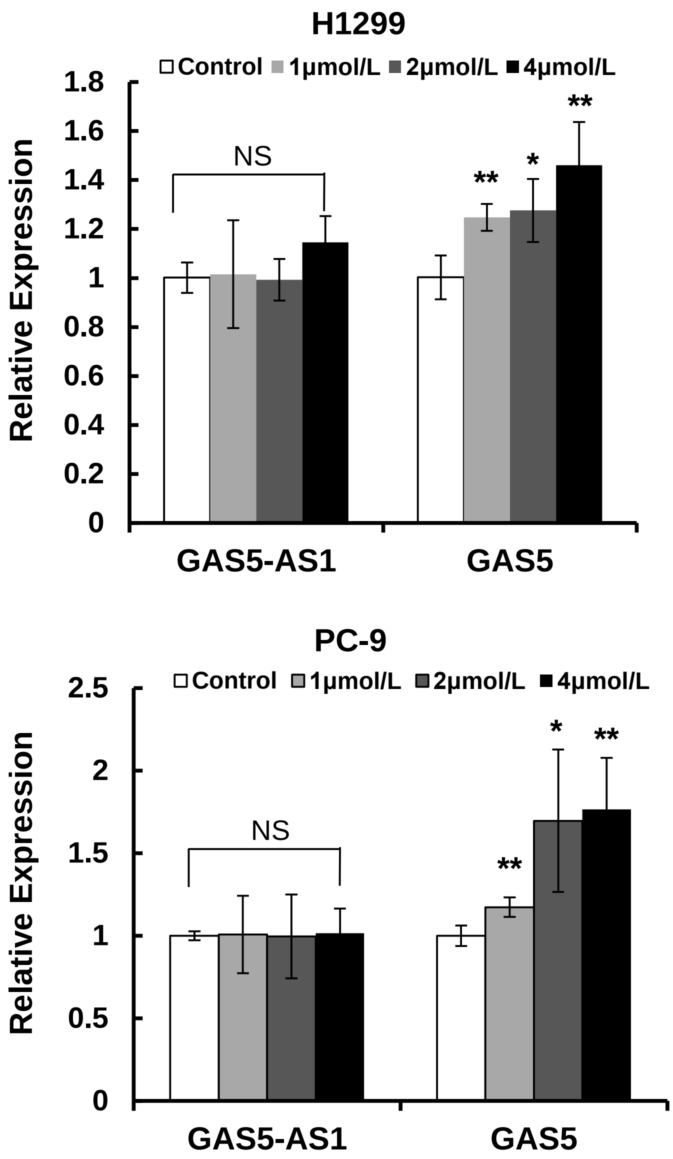

Supplementary Figure S1. H1299 or PC-9 cells untreated or treated with the indicated concentrations of decitabine for 48 h were collected and subjected to total RNA extraction. The expression levels of GAS5-AS1 and GAS5 were measured by qRT-PCR. **P*<0.05, ***P*<0.01, NS, not significant

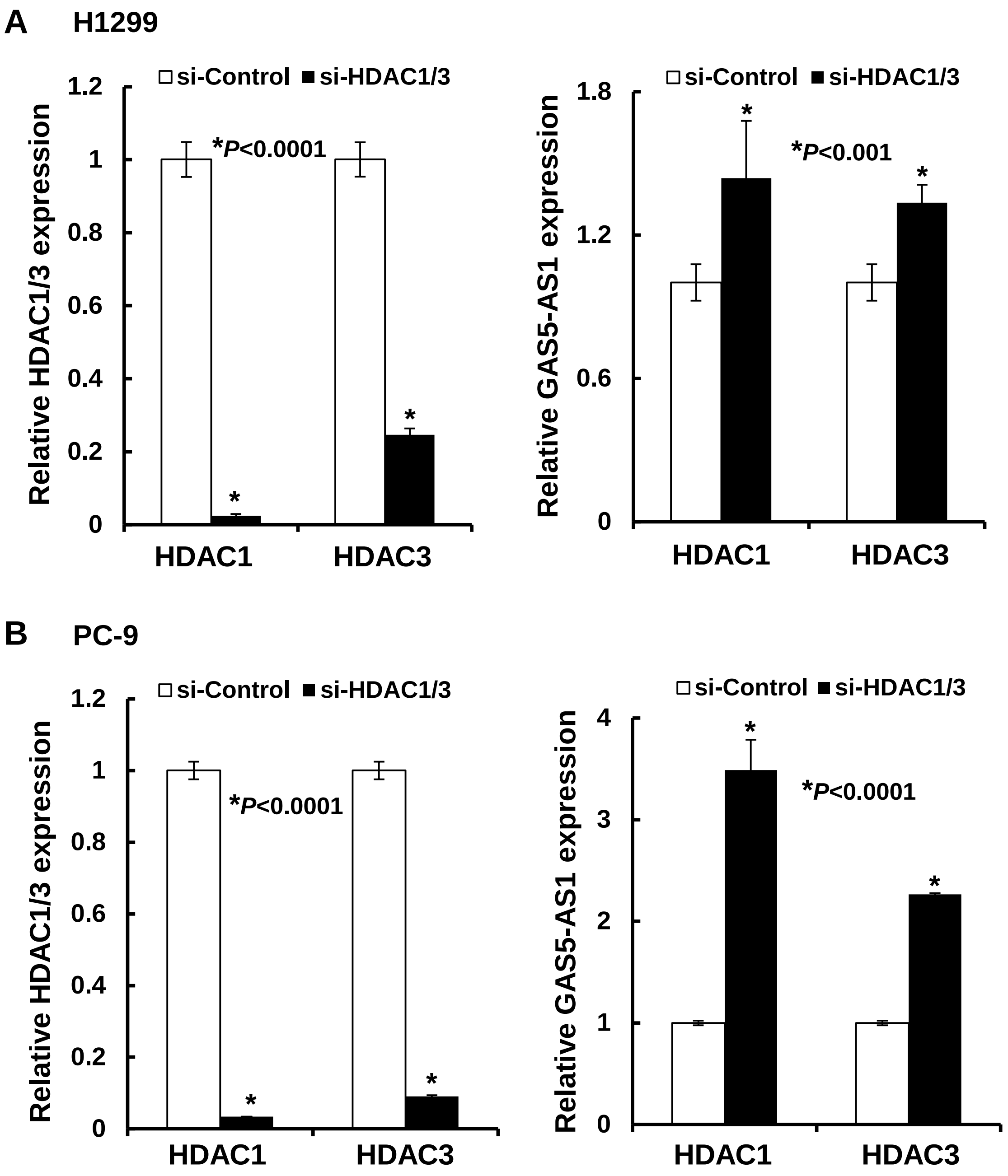

Supplementary Figure S2. Control siRNA (si-Control) or specific HDAC1 or HDAC3 siRNA (si-HDAC1/3) was transfected into H1299 or PC-9 cells for 48 hrs. The cells were then collected and subjected to the following studies. (A) qPCR analysis to measure the expression levels of HDAC1/3 and GAS5-AS1 in H1299 cells. (B) qPCR analysis to measure the expression levels of HDAC1/3 and GAS5-AS1 in PC-9 cells. Bars, SD. Data show a representative of three independent experiments.
